# Supplementary material for: Likely community transmission of COVID-19 infections between neighboring, persistent hotspots in Ontario, Canada
Source: F1000Res. 2022 Jul 21;10:1312. Originally published 2021 Dec 23. [Version 2] doi: 10.12688/f1000research.75891.2 (PMC9130760; doi:10.12688/f1000research.75891.2)
Supplement: Peer review history

# Peer review: Reviewer response for version 2

**DOI:** 10.5256/f1000research.136332.r144874

**Published:** 2022-08-08

**Authors:** Lorant Foldvary

**Affiliations:** 1 Department of Geodesy and Surveying, Associate Professor, Budapest University of Technology and Economics, Budapest, Hungary

Index as is.

Is the work clearly and accurately presented and does it cite the current literature?

Partly

If applicable, is the statistical analysis and its interpretation appropriate?

Yes

Are all the source data underlying the results available to ensure full reproducibility?

Partly

Is the study design appropriate and is the work technically sound?

Yes

Are the conclusions drawn adequately supported by the results?

Yes

Are sufficient details of methods and analysis provided to allow replication by others?

Yes

Reviewer Expertise:

geodesy, gravimetry, geoinformatics

I confirm that I have read this submission and believe that I have an appropriate level of expertise to confirm that it is of an acceptable scientific standard.

# Peer review: Reviewer response for version 1

**DOI:** 10.5256/f1000research.79823.r129171

**Published:** 2022-05-24

**Authors:** Ezra Gayawan

**Affiliations:** 1 Department of Statistics, Federal University of Technology, Akure, Nigeria

1. In the introduction, the authors, arguing against some geostatistical software, stated that “… however, these were not used in the present study because of their requirement to include disease background levels, which was not relevant for COVID-19.” I wonder what they really meant by “disease background levels” and how it restricts the use of the mentioned software including R for making geospatial analysis of COVID-19 data. As a fact, there are several of such analyses out there that used different methods and software particularly R. A Poisson geoadditive model, for instance, can be fitted to case count data on COVID-19 over time, which will generate some beautiful spatial maps without the need for any background disease as claimed.
2. The relationship between FSA and PC is not clear in the manuscript. This may be confusing for some readers, especially those not familiar with Canada. More detail is required in this aspect by clearly mentioning the benefits of why both FSA and PC were chosen rather than either one of them.  An illustrative graph showing the FSA and PC regions would be helpful.
3.  It is difficult to understand the illustration in Figure 1. A mathematical notation/formula should be used to support the illustration, clearly defining all the parameters involved in the formula.
4. The authors mention that "the software can be easily modified to accommodate other Canadian provinces and, with additional effort, other countries." However, there is no guide or link to a description of how the developed tool could be used and probably extended to other provinces or countries.
5. More details are required for the multinomial logistic model considered. Explicit details such as the response variable and the corresponding covariates should be clearly stated. If possible, the regression model should include stratification variables as covariates, such as categorized age, and "at-risk group" for example. The conclusion drawn from such analysis can be discussed in the Discussion session.  It is not clear whether the developed toolbox was used for the estimation of the multinomial regression model or the Directional Acyclic Graph Network.

Is the work clearly and accurately presented and does it cite the current literature?

Yes

If applicable, is the statistical analysis and its interpretation appropriate?

Yes

Are all the source data underlying the results available to ensure full reproducibility?

Yes

Is the study design appropriate and is the work technically sound?

Yes

Are the conclusions drawn adequately supported by the results?

Yes

Are sufficient details of methods and analysis provided to allow replication by others?

Partly

Reviewer Expertise:

NA

I confirm that I have read this submission and believe that I have an appropriate level of expertise to confirm that it is of an acceptable scientific standard, however I have significant reservations, as outlined above.

# Response

**Published:** 2022-07-21

**Authors:** Peter Rogan

**Affiliations:** University of Western Ontario, Canada

**Comment #1.** In the introduction, the authors, arguing against some geostatistical software, stated that “… however, these were not used in the present study because of their requirement to include disease background levels, which was not relevant for COVID-19.” I wonder what they really meant by “disease background levels” and how it restricts the use of the mentioned software including R for making geospatial analysis of COVID-19 data. As a fact, there are several of such analyses out there that used different methods and software particularly R. A Poisson geoadditive model, for instance, can be fitted to case count data on COVID-19 over time, which will generate some beautiful spatial maps without the need for any background disease as claimed.

**Response:**

 We appreciate this comment, in particular about the Poisson geoadditive model. Depending on which R script is used, analysis with R may or may not require background levels to be input. We have removed the citation to R, as suggested by another reviewer.

**Comment #2**. The relationship between FSA and PC is not clear in the manuscript. This may be confusing for some readers, especially those not familiar with Canada. More detail is required in this aspect by clearly mentioning the benefits of why both FSA and PC were chosen rather than either one of them.  An illustrative graph showing the FSA and PC regions would be helpful.

**Response:**

 To address the lack of familiarity on Canadian Forward Sortation Areas and Postal Codes, the sentence which introduces FSAs and PCs in the Introduction has been expanded. We also relate FSAs conceptually to U.S. zip codes: “distributed at the level of individual forward sortation areas (FSA) and postal codes (PCs; a smaller geographic segment within an FSA). Area-to-area analyses were performed on COVID-19 case data at the provincial level by FSA, a geographical unit similar to zip codes in the United States which can encompass hundreds of PCs.”  We have also added the following to Table 1: map images illustrating the PC boundaries of the municipalities of interest, a general Ontario map indicating the locations of each of these regions, the total number of FSAs and PCs in each region, and their total area of each.

 COVID-19 hotspot communities were often identified within FSAs. Prioritization for COVID-19 vaccinations (when supply was initially limited) was informed using hotspots identified at this level of granularity (Ontario Ministry of Health: COVID-19: Guidance for Prioritization of Phase 2 Populations for COVID-19 Vaccination. 2021). For this reason, we evaluated COVID-19 hotspots at this level of resolution. However, since the distributions of COVID-19 infections was often non-uniform in many FSAs (as we describe), this level of granularity was insufficient to fully elucidate its local distributions, and analysis of PCs was necessary.

 This possibility is mentioned in the Introduction (second paragraph): “Available case data analyzed has been typically aggregated at the municipal ^^or county level, ^^however higher resolution spatial analyses has been limited as finer granularity for the distribution of COVID-19 cases has often been lacking.“

 Subsequently, we generally describe FSAs and PCs. However, it was not obvious that PC-level COVID-19 case data produced a high level of geographic resolution that had been previously unavailable. We have therefore added the following at the end of this paragraph:: “Therefore, by evaluating COVID-19 case data at the PC level, we will be able to evaluate disease distributions at a high level of granularity.”

**Comment #3**. It is difficult to understand the illustration in Figure 1. A mathematical notation/formula should be used to support the illustration, clearly defining all the parameters involved in the formula.

**Response:**

 Formulation of the Getis-Ord local statistic is now presented in the third paragraph of the Introduction, with descriptions of each parameter within each equation, as requested.. We have also expanded the Figure 1 legend to describe how the weight function of these formulas differ depending on the spatial relationship being used. Weights of two features are equal in Fixed and K Nearest Neighbors, given that the two points are within a given distance metric (e.g. within 1km for Fixed, or is one of the K closest neighbors to the feature being evaluated for KNN; otherwise, *w _i,j_* = 0). The inverse distance spatial relationship, however, computes weight as the inverse Euclidean distance between two features being evaluated. This is calculated as *w _i,j_* = *d _i,j_^-β^* where *d _i,j_* is the Euclidean distance between two points and *β* is the inverse distance power variable ( *β* = 1 in this study). This is now provided in the legend of Figure 1.

**Comment #4**. The authors mention that "the software can be easily modified to accommodate other Canadian provinces and, with additional effort, other countries." However, there is no guide or link to a description of how the developed tool could be used and probably extended to other provinces or countries.

**Response:**

 We have added a section to the Geostatistical Epidemiology Toolbox user manual (in the Zenodo archive) which details the steps required for evaluation other Canadian Provinces or other countries. We have modified the original statement in the main manuscript to direct readers to the Geostatistical Epidemiology Toolbox user manual (which now contains a section on modifying the toolbox for other countries and Canadian provinces):“ While the toolbox was designed to evaluate COVID-19 case data in Ontario, the software can be easily modified to accommodate other Canadian provinces and, with additional effort, other countries (as described in the Geostatistical Epidemiology Toolbox user manual ^29^ ).”

 Using the toolbox to evaluate disease geostatistics in other Provinces is a relatively straightforward, requiring only alteration of a few lines of the main Python toolbox code (“GeostatisticalEpidemiologyToolbox_CytoGnomix.pyt”; see Toolbox guide sub-section “Evaluating Other Canadian Provinces” within the Zenodo archive file “Software.GeostatisticalEpidemiologyToolbox.zip”).

 Modifying the Toolbox to evaluate hotspots and interpolation in other countries would be more involved ( “Evaluating Other Countries” section of the manual). The structure of the shape files that define the regions equivalent to an FSA or a postal code in other countries will likely follow a different naming convention (for example, county subdivisions and zip codes in the United States). We now indicate in the Toolbox user manual which column names used by the Toolbox are most likely to require modification ( ‘PROV’ and ‘PRNAME’). Since it is not possible to generalize the structure of every shape file defining geographic elements of other countries, potential users should consider contacting us for assistance.

**Comment #5**. More details are required for the multinomial logistic model considered. Explicit details such as the response variable and the corresponding covariates should be clearly stated. If possible, the regression model should include stratification variables as covariates, such as categorized age, and "at-risk group" for example. The conclusion drawn from such analysis can be discussed in the Discussion session.  It is not clear whether the developed toolbox was used for the estimation of the multinomial regression model or the Directional Acyclic Graph Network.

**Response**:

 Postal codes were identified as a high-case cluster (by Cluster and Outlier analysis) over a period of >3 consecutive days (i.e., PC “streaks”), and established which of these streaks occurred in two neighboring PCs in close succession. We noticed that the number of cases reported in each paired streak, the interval of time separating each streak, and the physical distance between the PCs might be related. To establish this, we derived multinomial logistic regression models based on these factors, in which the total number of cases within the paired streaks was defined as the response variable. The relationship between the number of cases between streaks of pairs of PCs in close proximity and the time separating the occurrence of those two streaks was significant.

 We now provide the R code used to derive these multinomial logistic regression models (“Streak_Analysis_using_Multinomial_Logistic_Regression_Models.Rmd”) as a new section within the Zenodo archive (“Software.Additional_Programs_for_Cluster_Outlier_Streak_Identification_and_Pairing.zip”). The paragraph describing the multinomial logistic regression model (third paragraph of the Results has been updated to reflect this change: “The distribution of and relationships between clustered PCs with persistent COVID-19 cases”). This section has been revised to describe the software used to derive the model (including the name of the script and a citation of the Zenodo archive), and explicitly states which of the three variables was used as the response variable: The input data are also included in the archive.

 The description now states: “To assess the significance of these results, a multinomial logistic regression model was derived in R (“Streak_Analysis_using_Multinomial_Logistic_Regression_Models.Rmd” which uses the *multinom* function of the *nnet* package; available in Zenodo archive ^29 ^), evaluating (1) the total number of cases in each pair of PCs over the course of both streaks (classified as either <25, between 25 and 49, or ≥50 cases **; **the response variable of the regression), (2) the duration … ”

 This analysis was not performed for stratified case data because it considerably decreases the number of high-case clusters detected per class (Table 1). This would result in far fewer and shorter PC “streaks”, and the lower statistical power would make analysis would less meaningful.

 The Geostatistical Epidemiology Toolbox was developed to perform and visualize geostatistical analyses through the ArcMap graphical user interface using ArcPy (which enables Python programs to access ArcGIS’s geoprocessing tools and extensions). The directional acyclic graph networks, as well as the regression, were performed outside of this environment (using MatLab and R, respectively). Furthermore, these techniques examined a subset of our results. Thus, the toolbox does not and can not perform either task.

# Peer review: Reviewer response for version 1

**DOI:** 10.5256/f1000research.79823.r129170

**Published:** 2022-04-08

**Authors:** Christine Tedijanto

**Affiliations:** 1 Francis I. Proctor Foundation, University of California, San Francisco, San Francisco, CA, USA

The authors present a thorough geospatial analysis of COVID-19 cases in Ontario, Canada. In this work, they explore a number of different global and local clustering methods in order to demonstrate (1) the importance of higher resolution data to monitor and control COVID-19 and (2) potential transmission between hotspots. They find alignment between the different clustering approaches and evidence of neighboring areas experiencing high case burdens at similar times, implying transmission between communities. In general, I thought the analyses were interesting, but the work could be made more impactful by streamlining the text and focusing on a clearer through line.

  

1. For readers who may not be familiar with Ontario, FSAs, and PCs, it may be helpful to include additional background material such as (1) a map of Ontario showing the location of the six municipalities (and FSAs, if possible) of interest, (2) the number of FSAs/PCs and their approximate population sizes, and (3) overall case counts in your municipalities of interest in each of the waves and interwave periods.
2. The motivation behind the subgroup analyses (sex, age, health conditions) was not clear to me. It seems like clusters within each subgroup would tend to appear simply where the highest number of individuals in each subgroup reside, and I’m not sure we would expect the transmission to occur along subgroup lines (for example, we might expect younger family members to transmit to older ones rather than transmission occurring between older individuals). Please explain the reasoning and implications of this analysis further; it may be more suitable in the Extended Data as it seems less relevant to the overall conclusions of the manuscript.
3. I found it difficult to distinguish between the space-time Gi* and the “streak” analyses. How do they relate to one another? Is there any additional information gained from the space-time Gi* analysis?
4. Please describe in more detail the approach that was used to evaluate the kriging and semivariogram models. Why do you think the models performed differently in Toronto compared to other cities? In addition, please clarify – were PCs with 0 cases also excluded as neighbors (i.e., were neighbors in the kriging analysis restricted to neighbors with cases >=1)?
5. Is there a way to incorporate uncertainty in the kriging analysis? I would also be cautious about using the term “significant” to describe areas with interpolated counts >5, as this may be mistaken for statistical significance.
6. I thought the graphs in Figure 8 were a bit challenging to interpret. Visually, it may help to arrange the points similarly to how they are geographically located and/or to change the length of the edges in proportion to the duration separating adjacent pairs (the current label).
7. I would like further discussion about how the authors see these analyses being applied, especially in real-time. For example, the first paragraph of the Discussion says that this method “enabled early detection of disease clusters” – please provide additional evidence of this from the analyses. Additionally, based on the authors’ experience of applying many different methods, are there specific ones that the authors would recommend for real-time use (or public health practice in general)?

**Minor comments:**

1. In the second paragraph of the introduction, the authors include R in a list of tools that were not used because they require background levels, but R is very general software with packages that may be used without disease background levels. I would consider removing it from this list.
2. In my opinion, some sections of the methods may be moved to the Extended Data (e.g. paragraphs beginning “Additional ArcPy scripts…” and “These processes were then integrated…”) for conciseness. Tables 1 and 2 may also be better suited to the Extended Data.
3. Please clarify the paragraph about FSAs with case counts <5 – was it possible to analyze these FSAs without masking, just within a separate computing environment? It was not clear to me whether they were masked or not in the final analysis.
4. In the fourth paragraph of the results, why were comparisons between kriging and the cluster/outlier methods restricted to March 26 to December 28, 2020?
5. In Figure 3B, what do the blue crosses represent? I thought they represented the observed data, in which case I would have expected the same blue crosses in the top and bottom panel. Please clarify.
6. Please provide additional information on the “relative density” metric in Figure 4. Is there a reason this particular area and time were highlighted in this figure? It may be interesting to show a hotspot that occurred during a wave rather than in a time window with low disease burden.
7. If possible, please combine all resources into one repository.
8. I did not see underlying data in any of the linked repositories. If the underlying data cannot be publicly shared, please provide a statement describing how the data may be accessed.

Is the work clearly and accurately presented and does it cite the current literature?

Partly

If applicable, is the statistical analysis and its interpretation appropriate?

Partly

Are all the source data underlying the results available to ensure full reproducibility?

Partly

Is the study design appropriate and is the work technically sound?

Yes

Are the conclusions drawn adequately supported by the results?

Partly

Are sufficient details of methods and analysis provided to allow replication by others?

Yes

Reviewer Expertise:

Infectious disease epidemiology

I confirm that I have read this submission and believe that I have an appropriate level of expertise to confirm that it is of an acceptable scientific standard, however I have significant reservations, as outlined above.

# Response

**Published:** 2022-07-21

**Authors:** Peter Rogan

**Affiliations:** University of Western Ontario, Canada

**Major Comment #1:**

 ...it may be helpful to include additional background material such as (1) a map of Ontario showing the location of the six municipalities (and FSAs, if possible) of interest, (2) the number of FSAs/PCs and their approximate population sizes, and (3) overall case counts in your municipalities of interest in each of the waves and interwave periods.

**Response:**

 We agree that including regional statistics and maps of the areas being studied would be beneficial to the manuscript. We have therefore chosen to append this information to Table 1A of the manuscript, as the structure of the table was well suited for it. The first column of Table 1 includes a map of lower Ontario with the location of each region of interest, as well as maps of each region indicating their boundaries. We also include the population, the area (km ^2^) and the number of FSAs and PCs in each region analyzed.

 We have also added a new column which gives an estimate of the number of cases present in each region (the sum of cases from individual FSAs associated with the region of interest), divided by waves. These case numbers are estimated, since our contract with ICES has ended, and they have removed our access to actual case data. Thus, any dates in which case data was masked (i.e. the FSA had between 1-5 cases on a particular date) were considered to be a single case by this calculation (which is indicated in the footer of Table 1).

**Major Comment #2:**

 The motivation behind the subgroup analyses (sex, age, health conditions) was not clear to me. It seems like clusters within each subgroup would tend to appear simply where the highest number of individuals in each subgroup reside, and I’m not sure we would expect the transmission to occur along subgroup lines (for example, we might expect younger family members to transmit to older ones rather than transmission occurring between older individuals). Please explain the reasoning and implications of this analysis further; it may be more suitable in the Extended Data as it seems less relevant to the overall conclusions of the manuscript.

**Response:**

 The identification of COVID-19 clusters according to these subgroups was specifically requested by the Ontario Ministry of Health. There was interest in identifying differences in how COVID-19 cases were distributed across our regions of interest. This has merit since clustering of a particular subgroup was often correlated with known events occurring in those locations, e.g. high-case clusters of COVID-19 in predominantly young males in rural regions of Windsor/Essex reflecting outbreaks in migrant farm worker communities at this time). We have not moved this analysis (including Table 1) to the extended data.

 The analysis we describe does not imply that transmission is occurring between or within subgroups. Nonetheless, the distribution of cases by subgroup was of particular interest to the Ontario Ministry of Health. It did not reflect the geographic bias in the residence of subgroup members. We have therefore added the following to the end of the fourth paragraph of Results subsection “Wave 1” (page 17, paragraph 1 without markup): “This stratified analysis itself does not provide insight into the mode of COVID-19 transmission, that is, the development of a hot spot consisting of a particular subgroup does not indicate that viral transmission occurred exclusively between members of the particular subgroup.”

**Major Comment #3:**

 I found it difficult to distinguish between the space-time Gi* and the “streak” analyses. How do they relate to one another? Is there any additional information gained from the space-time Gi* analysis?

**Response**: 

 Space-time Gi* analysis and Cluster and Outlier ‘streaks’ are indeed two separate analyses.

 Results using space-time Gi* analysis (built-in to ArcGIS) is provided at the start of the Results. We found that space-time Gi* analysis was generally uninformative, as the analysis called nearly all regions with high numbers of cases (>10 at the FSA level) as statistically significant (>90% in most waves). Therefore, we did not pursue it further. However, this was not explicitly stated, so we have added the following statement to the aforementioned Results paragraph: “As the vast majority of regions were flagged by this approach (>90%), we did not gain any insight from this space-time analysis.”

 We then investigated temporal trends using  Cluster and Outlier analyses of “streaks” of consecutive days that particular locates were deemed hotspots. analysis  This is a form of “space-time” analysis, but is completely independent of any functional tests built into ArcGIS. We aren't aware of any prior description of this method. We have modified the text to clarify that this was completely separate from space-time Gi* analysis (page 14, paragraph 2): “Instead, we evaluated temporal trends in the distribution of COVID-19 cases and identified potential transmission using Cluster and Outlier analysis of PCs that persisted across a consecutive series of dates (referred to as high-case cluster ‘streaks’; described later in the Results section).”

**Major Comment #4:**

1. Please describe in more detail the approach that was used to evaluate the kriging and semivariogram models.
2. Why do you think the models performed differently in Toronto compared to other cities?
3. In addition, please clarify – were PCs with 0 cases also excluded as neighbors (i.e., were neighbors in the kriging analysis restricted to neighbors with cases >=1)?

**Response:**

 (A) In the second paragraph of Methods subsection “Empirical Bayesian Kriging analysis”, we describe our reasoning for prioritizing the Power and TPS semivariograms for low/medium and high case density regions, respectively. Some detail was excluded due to the overall length of the manuscript. To further elaborate, we chose to evaluate all available kriging semivariogram options in ArcGIS in moderate (London, ON) and high (Toronto, ON) density regions across several dates in Waves 1 and 2 of the COVID-19 pandemic. Each semivariogram was tested under various setting conditions (sector type [1 or 4 sectors], search radius [the optimal radius determined by ArcGIS software, that value halved and that value doubled], neighborhood type [smooth or standard] and the number of neighbors considered for each location [5, 10, 15]). The kriging contours generated were converted to polygons in order to perform an ‘intersect’ in ArcGIS between these contours and PCs, and from this intersection, the values interpolated by kriging were compared to true case counts.

 The Power semivariogram generally modeled counts better in low and medium-density municipal regions. Although TPS often performed well, it produced artifact contours in regions without cases, as described in the Methods (“TPS, in some instances, generated kriging artifacts that did not correlate with known hotspots”). However, the Power semivariogram model was sometimes inferior to TPS, with PCs having high case counts were being missed. Thus, we used TPS in Toronto, considering only 5 neighbors which minimized the kriging artifacts without sacrificing accuracy. This observation was supported by a chi-square test, whereby kriging interpolation with the TPS semivariogram utilizing ≥10 neighbors was frequently significantly different than ground truth (e.g. 72% of dates in Wave 2 were significantly different [p<0.05] when considering 10 neighbors) This did not happen when considering 5 neighbors (p>0.05 for all dates in Waves 1 and 2). This analysis justifies our selection for the number of neighbors parameter when performing kriging, and this analysis is now briefly described in the manuscript: “Using 5 neighboring locations for interpolation minimized these artifacts without affecting the contour map. However, with 10 neighbors, interpolation accuracy was affected according to a chi-square test (e.g., p < 0.05 for the 103 days in Wave 2).”

 With similar tests, we found that the default kriging parameters (other than neighbor count) were optimal (or lead to equivalent results) regardless of semivariogram used. Multiple kriging parameters are used to define a search neighborhood, the window which delineates which measured locations will and will not be included to predict the value of a region. One such parameter divides this search window into multiple sectors. However, utilizing four sectors led to poorer interpolation of true case counts by chi-square test (significantly different [p<0.05] for 37% of dates in Wave 1 and 82% of dates in Wave 2). Thus, a single sector was used. These parameters are now mentioned in the Methods section: “All other EBK parameters used were defaulted to those provided by the ArcGIS Advanced Geostatistics toolbox (Sector Type: 1 sector, Neighborhood Type: Standard, Search Radius: 1).”

 The use of TPS in Toronto is now justified. Postal codes with >5 cases are interpolated as such (≥5-10 cases) over twice as often with TPS relative to Power (991 vs 414, respectively). Furthermore, the root mean square error of case count to interpolated case range of PCs with > 5 cases is higher using the Power semivariogram (6.4 with Power, 5.4 with TPS), indicating that TPS outperforms Power at interpolating case counts. A chi-square test comparing the interpolation of daily Toronto case counts using the TPS and Power semivariograms similarly showed that while use of the Power semivariogram was never significantly different from true case counts, the TPS semivariogram better matched ground truth (p-value TPS > p-value Power for 85% and 78% of dates in Waves 1 and 2, respectively).

Similarity of Interpolation to True Cases by Chi-Square Test: Kriging utilizing the Power and TPS Semivariogram

**Days with Greater Similarity [> p-val] to actual cases             Days (≥1 PC with ≥ 6 Cases)**

*p _TPS_ > p _POWER_***__** *p _POWER_ > p _TPS      _ p _TPS_ = p _POWER_*

*Wave 1:*

 60                               2                            9                                      71

*Wave 2:*

 112                            11                           20                                    143

 All six municipalities were evaluated with both the Power and TPS semivariograms across all dates evaluated, the output of which is available in the Zenodo archive.

 (B) We believe that semivariogram performance in Toronto was due to its far higher population frequency and density relative to the other areas analyzed. Toronto has the highest density of postal codes (75.3 PCs per km ^2^) compared to the other regions investigated (Hamilton: 13.0 PCs per km ^2^; Kitchener/Waterloo: 42.0 PCs per km ^2^; London: 23.9 PCs per km ^2^; Ottawa: 7.9 PCs per km ^2^; Windsor/Essex: 6.0 PCs per km ^2^). While the PC density of Kitchener/Waterloo is somewhat comparable to Toronto, the population density of Kitchener/Waterloo is 2.5x smaller. Furthermore, the number of COVID-19 cases experienced between the two regions were far different, with Toronto having nearly 15-fold more cases compared to Kitchener/Waterloo (see Table 1).

 Toronto exhibits far more PCs with cases than the other regions tested on most dates. The majority of these PCs have cases between 1-5 (<1% of PCs in Toronto had >5 cases across all dates analyzed). This pushes the interpolated case counts downwards (similar to how zero-case PCs depressed kriging values; see next section). Since the “Power” semivariogram is a parabolic mathematical representation of cases in the region, a large amount of low-case PCs may ‘smooth’ any contour peaks in the region. Since TPS semivariograms attempt to pass through binned data points exactly, these peaks are not lost. Thus, the true case count of high case areas are better represented within the kriging contour.

 Kriging analysis was performed on COVID-19 case data that did not include PCs with zero cases. In the first paragraph of Methods subsection “Empirical Bayesian Kriging analysis”, we state: “Only PCs with ≥1 case were included in this analysis, as locations without confirmed cases were found to severely depress kriging signals.”

 PCs with zero case counts are more frequent, even on dates where PCs with cases were at its maximum (e.g. from April 12 to the 14, 2021, 2377 PCs in Toronto had ≥1 case, which is only 5% of the total number of PCs in this region [2.4 to 3.8% for the other 5 regions of interest). These PCs were so pervasive that they depressed the interpolated values of actual cases in PCs with cases. The case count interpolated by kriging was never representative of (i.e. biased against) actual case counts when zero case PCs were included in our testing. We modified the relevant (above) to further explain this: “Only PCs with ≥1 case were included in this analysis, as locations without confirmed cases greatly outnumbered PCs with cases (≥95% of PCs on any given date evaluated) leading to the severe depression of kriging signals.”

**Major Comment #5:**

 Is there a way to incorporate uncertainty in the kriging analysis? I would also be cautious about using the term “significant” to describe areas with interpolated counts >5, as this may be mistaken for statistical significance.

**Response:**

 The ArcGIS software is capable of determining uncertainty when performing Kriging ( https://desktop.arcgis.com/en/arcmap/10.5/extensions/geostatistical-analyst/kriging-in-geostatistical-analyst.htm )​​​​​ with standard error maps ( https://desktop.arcgis.com/en/arcmap/latest/extensions/geostatistical-analyst/using-ordinary-kriging-to-create-a-prediction-standard-error-map.htm). We no longer have access to the ICES computer system to generate such plots. Furthermore, The ‘layer’ files are linked to the input used to generate the kriging layer which is no longer available to us.

 The main text has been edited to remove instances of the term “significant” for regions interpolated by kriging consisting of >5 case counts. This was changed to “region of interest”, “localized hotspot”, or avoided. The name of Supplemental Figure S3 was also changed.

**Major Comment #6:**

 ...graphs in Figure 8 were a bit challenging to interpret. Visually, it may help to arrange the points similarly to how they are geographically located and/or to change the length of the edges in proportion to the duration separating adjacent pairs (the current label).

**Response**: 

 The directional network graphs in Figure 8 were created with the MatLab *digraph *function. *digraph* does not provide any option to incorporate the recommended changes. The order of data points and edge length are automatically specified. Our only option was to label each path with distance, number of days separating pairs of neighboring streaks, PC identifiers, etc. The maps accompanying the graphs provide a geographic context for each network. Figure 8 and Extended Data Tables S9 and S10 remain unchanged.

**Major Comment #7:**

 I would like further discussion about how the authors see these analyses being applied, especially in real-time. For example, the first paragraph of the Discussion says that this method “enabled early detection of disease clusters” – please provide additional evidence of this from the analyses. Additionally, based on the authors’ experience of applying many different methods, are there specific ones that the authors would recommend for real-time use (or public health practice in general)?

**Response:**

 We provide an example within which the FSA N5Y in London Ontario was identified as a region of interest (>5 cases interpolated) by kriging, one week before it was publicized (Figure 6). To provide further evidence of early detection, additional examples (in a new supplementary figure S3A and S3B) of identified localized hotspots prior to public announcement are described:  “Localized hotspots within the FSA M9P were observed as early as March 31 ^st^, 2020 however the FSA was not flagged by Gi* analysis until April 6 ^th^, 2020 (Extended data, ^^Section 1 - Figure S3A). The localized hotspot covers the Village of Humber Heights Retirement Home in Toronto, which was reported to have a COVID-19 outbreak on April 12 ^th^, 13 days after the localized hotspot was first detected by kriging. “ “For example, localized hotspots in FSA M9V were identified sporadically, and the interpolated value does not always match total cases (Extended data, ^^Section 1 - Figure S3B). The localized hot spots cover the PC M9V 5B5 starting from April 4 ^th^. Outbreaks in the long-term care home within this PC (Humber Valley Terrace long-term care home in Toronto) were reported on April 16 ^th^, 2020. This FSA was not flagged by Gi* analysis until April 19 ^th ^(Extended data, ^^Section 1 - Figure S3B).”

 There were several instances where kriging identified a localized hotspot prior to public declaration within the same region. These was not found by either  Gi* analysis or Cluster and Outlier analysis. While kriging analysis requires more time than these other statistics to generate results (minutes vs seconds per hotspot), software automation on high-powered systems at provincial and municipal scale improved performance of these processes. In April 2021, the Ontario Ministry of Health requested that we perform a full geostatistical analysis of COVID-19 in four municipal regions [London, Ottawa, Toronto and Windsor/Essex] from January and March 2021. We returned the results, including an evaluation of localized hotspots with kriging, within 5 days (see Discussion, paragraph 7). The “Streak” analysis method (based on results from the Cluster and Outlier analysis tool) was only used to trace disease spread; this was a retroactive analysis to identify neighboring, persistent, and overlapping COVID-19 clusters. We believe that additional work is required to prove the validity of use of this approach pro-actively.

 Early hot spot detection is predicated on the availability of current and complete case data. Except for holiday periods, COVID-19 case data was preprocessed and updated on a weekly basis at ICES. As previously published, the number of cases on the final 3 days of the prior dataset was updated and usually increased in the subsequent release (additional testing data was added to those dates), especially in rural regions where testing was subject to adjustment. We have therefore added the following to the Discussion: “… and enabled early detection of disease clusters, which could be exploited to prevent or limit further spread at or close to these hotspots (provided complete and up-to-date COVID-19 case data is made available in a timely manner).”

**Minor Comment #1:**

 In the second paragraph of the introduction, the authors include R in a list of tools that were not used because they require background levels, but R is very general software with packages that may be used without disease background levels. I would consider removing it from this list.

**Response:**

 Geostatistical epidemiology has usually required pre-existing background levels of disease, which is not relevant at the beginning of the COVID-19 pandemic. R and the majority of available epidemiology packages include background level adjustment. While some R packages may permit background to be ignored, we have removed R  from the software listed.

**Minor Comment #2:**

 In my opinion, some sections of the methods may be moved to the Extended Data (e.g. paragraphs beginning “Additional ArcPy scripts…” and “These processes were then integrated…”) for conciseness. Tables 1 and 2 may also be better suited to the Extended Data.

**Response:**

 Table 2 has been moved to the Extended Data, as requested. Table 1 remains, as previously discussed (Reviewer #2, Major Comment #2). The paragraph beginning “These processes were then integrated…” contains our description of the Geostatistical Epidemiology Toolbox, which is important for reproducibility and portability of the methods we describe. We highlight this in the paper, and F1000Research requires that the software be available as a condition of publication. We have reduced its length rather than  removed it. The deleted, relevant information can now be found within the User Manual accompanying the toolbox (“UserManual_GeostatisticalEpidemiologyToolbox_CytoGnomix.pdf”) The paragraph beginning with “Additional ArcPy scripts…” has been moved to the Zenodo archive (“Documentation_for_Each_Section_of_Zenodo_Archive.docx”.

**Minor Comment #3:**

 Please clarify the paragraph about FSAs with case counts <5 – was it possible to analyze these FSAs without masking, just within a separate computing environment? It was not clear to me whether they were masked or not in the final analysis.

**Response:**

 All analyses described using unmasked true case count data within the ICES environment, the only exception being the Gi* analysis that was performed to assess the impact of masking on the geostatistical results (described in the fourth paragraph of the Methods, where we found that masking significantly affected results: “We concluded that errors introduced by masking count values precluded analysis of masked data and required all work to be carried out within the RAE computing environment.”

 We now explicitly state that no other geostatistical analysis described in this study used masked data, with the following statement in the Methods: “All geostatistical analyses presented in this study were performed against unmasked, true case counts.”   We also modified an earlier statement in the same paragraph regarding ICES stipulation that we mask FSAs/PCs with 1-5 cases to clarify that this practice applied only to data export:  “To ensure patient privacy, ICES stipulated that case counts could not be exported from the ICES system unless FSAs and/or PCs exhibiting between 1 and 5 daily COVID-19 cases were masked.”  The following was also added to the Results (first para.): “Changes in the distribution of COVID-19 infections over time were evaluated through geostatistical analysis of true (unmasked) case counts within both FSAs and PCs.”

**Minor Comment #4:**

 In the fourth paragraph of the results, why were comparisons between kriging and the cluster/outlier methods restricted to March 26 to December 28, 2020?

**Response:**

 The comparison between kriging and Cluster and Outlier analysis was part of a feasibility analysis (performed in January 2021) to compare the two methods. Expanding this analysis was unlikely to provide novel insights. We prioritized our limited access time to the data and computing environment to perform other analyses.

**Minor Comment #5:**

 In Figure 3B, what do the blue crosses represent? I thought they represented the observed data, in which case I would have expected the same blue crosses in the top and bottom panel. Please clarify.

**Response:**

 Blue crosses are bins of paired locations, grouped by distance and direction (as described in the Figure 3 description). How these bins are grouped by ArcGIS software is indeed different whether the Power or TPS semivariogram is being used. Additionally, how data points are binned can regionally differ, as the values and distances between points vary from location to location.

 However, the two semivariograms from Figure 3B were not associated with each other. The original purpose of Figure 3B was to  provide examples of “Power” and “TPS” semivariograms derived from this data. While not implicitly stated in the figure legend, we see now that how the inferrence of a relationship between the two semivariograms could be made. Figure 3B has therefore been updated with semivariograms that are representative of the kriging map presented in Figure 3A. The binned blue crosses for the “Power” and “TPS” semivariograms are similar, although not identical (for the reasons given earlier).

**Minor Comment #6:**

 Please provide additional information on the “relative density” metric in Figure 4. Is there a reason this particular area and time were highlighted in this figure? It may be interesting to show a hotspot that occurred during a wave rather than in a time window with low disease burden.

**Response:**

 Figure 4 represents a region where COVID-19 cases were highly dispersed over a period of time. There are indeed likely other additional regions that could have been used, however the example selected is indeed interesting and worthy of discussion. The time window (September 10 ^th^ to the 29 ^th^, 2020) spans the very end of Interwave 1 (September 24 ^th^, 2020), leading into Wave 2, a time where the frequency of cases was rapidly increasing. The frequency of dates in which this FSA was called significant by Gi* analysis was similarly increasing. We therefore do not feel that replacing this figure for another example would be beneficial.

**Minor Comment #7:**

 If possible, please combine all resources into one repository.

**Response:**

 We have combined the data and software Zenodo archives into a single archive (titled ‘Data and Software Archive for "Likely community transmission of COVID-19 infections between neighboring, persistent hotspots in Ontario, Canada"’). All references to the Zenodo archive within the main text now point to this archive.

**Minor Comment #8:**

 I did not see underlying data in any of the linked repositories. If the underlying data cannot be publicly shared, please provide a statement describing how the data may be accessed.

**Response:**

 The underlying data can be found in the file "Section_3.All-Data_Files.Kriging_GiStar_Local_and_GlobalMorans.2020_2021.zip' in the Zenodo archive ( https://zenodo.org/record/5585812#.YtkdD3bMI2w). It contains not only output from our geostatistical analyses, but other Ontario COVID-19 case data as well (where cases between 1-5 are masked, as required). This is mentioned in the “Data Availability” section of the manuscript:

 Original statement: “Sections 3 and 4 contains the data files containing and the map image files displaying the underlying statistics from the various geostatistical analyses performed in this study.”

 This has now been clarified to indicate that Section 3 contains the data files, while Section 4 contains map images: “Sections 3 and 4 respectively consist of data files containing and map image files displaying the underlying statistics from the various geostatistical analyses performed in this study.”

 We also modified the “Extended Data” subsection that describes Section 3, changing the term “output files” to “underlying data” to make the location of the underlying data clearer.

# Peer review: Reviewer response for version 1

**DOI:** 10.5256/f1000research.79823.r129168

**Published:** 2022-04-05

**Authors:** Lorant Foldvary

**Affiliations:** 1 Department of Geodesy and Surveying, Associate Professor, Budapest University of Technology and Economics, Budapest, Hungary

Brief Report:

**Q: Is the work clearly and accurately presented and does it cite the current literature?**

 A: The work is clearly and accurately presented, but the citation of the relevant literature is incomplete. For example, on page 3, in the Introduction: "Various geostatistical analyses have been used in the surveillance of COVID-19 spread across the globe" 

 Relevant examples are missing such as Földváry (2021) ^1^ but most essential referring to the solution at Johns Hopkins University. ^2^.

**Q: Is the study design appropriate and does the work have academic merit?**

 Yes, the study design is fine, the academic merit, and the scientific value is notably high. 

**Q: Are sufficient details of methods and analysis provided to allow replication by others?**

 A: The used methods are fine, this is the strength of the study. Since the analysis is performed on commercially available software, the study might be replicated if the used data is accessible.

**Q: If applicable, is the statistical analysis and its interpretation appropriate?**

 A: Yes, the statistical analysis are fine, the interpretation of the results is reasonable!

**Q: Are all the source data underlying the results available to ensure full reproducibility?**

 A: The information on the source data in the manuscript is a bit confusing. Accordingly, improvement of this section is suggested (see the next paragraph). The data and software availability is provided, this content seems to be fine, although the reviewer has no capacity to check on the full reproducibility. 

 On the source data: 

 At distinct locations of the text some information on the data is provided, e.g. in the Geostatistical analysis subchapter of the Methods chapter (page 4) says: "COVID-19 cases from March 26th, 2020 to June 25th, 2021 at the FSA (Ontario-wide) and PC-level (for 6 populated areas: Hamilton, Kitchener/Waterloo, London Ottawa, Toronto, and Windsor, including Essex county) were evaluated using ArcGIS Desktop 10.7 [ESRI] with the Geostatistical Analyst extension". It is followed by the first information on the data (page 6, the same subchapter) by indicating that "COVID-19 testing data compiled by ICES contained metadata on tested individuals, such as gender, age, and relevant predicate health conditions", and only later, at the subchapter titled Preparation of Ontario COVID-19 case data from ICES (page 7), we get the introduction of the data source. Somehow the introduction of the data source should be provided more consistently, maybe in a separate chapter before "Methods" titled "Data". The importance of the data for any geostatistical study is essential since the quality and the reliability of the data validates the conclusions.

**Q: Are the conclusions drawn adequately supported by the results?**

 A: Conclusions and interpretations of the results are adequately drawn.

**Additional detailed comment:**

 Page 6, Methods: "COVID-19 testing data compiled by ICES contained metadata on tested individuals, such as gender, age, and relevant predicate health conditions." 

 This is the first appearance of the abbreviation ICES, please provide here the full name of the organization!

Is the work clearly and accurately presented and does it cite the current literature?

Partly

If applicable, is the statistical analysis and its interpretation appropriate?

Yes

Are all the source data underlying the results available to ensure full reproducibility?

Partly

Is the study design appropriate and is the work technically sound?

Yes

Are the conclusions drawn adequately supported by the results?

Yes

Are sufficient details of methods and analysis provided to allow replication by others?

Yes

Reviewer Expertise:

geodesy, gravimetry, geoinformatics

I confirm that I have read this submission and believe that I have an appropriate level of expertise to confirm that it is of an acceptable scientific standard.

## References

[1] : Geostatistical Investigations on the Spread of COVID-19. *International Journal of Geoinformatics* .2021; 10.52939/ijg.v17i1.1713 75-84 10.52939/ijg.v17i1.1713

[2] : An interactive web-based dashboard to track COVID-19 in real time. *The Lancet Infectious Diseases* .2020;20(5) : 10.1016/S1473-3099(20)30120-1 533-534 10.1016/S1473-3099(20)30120-1 32087114PMC7159018

# Response

**Published:** 2022-07-21

**Authors:** Peter Rogan

**Affiliations:** University of Western Ontario, Canada

**Comment #1:**

 The work is clearly and accurately presented, but the citation of the relevant literature is incomplete. For example, on page 3, in the Introduction: "Various geostatistical analyses have been used in the surveillance of COVID-19 spread across the globe" 

 Relevant examples are missing such as Földváry (2021) but most essential referring to the solution at Johns Hopkins University (Dong et al., 2020).

**Response:**

 We now cite Földváry *et al.* (2021) in the Introduction of our manuscript, as suggested by the reviewer. We have omitted Dong *et al.* (2020), as the web resource described in this study simply displays the location of official COVID-19 counts across the world. This paper focuses on literature that performs geostatistical analyses of the distribution of COVID-19 cases beyond simple counts (e.g. disease spread directionality).

**Comment #2:**

 The information on the source data in the manuscript is a bit confusing. Accordingly, improvement of this section is suggested (see the next paragraph). The data and software availability is provided, this content seems to be fine, although the reviewer has no capacity to check on the full reproducibility. 

 On the source data: 

 At distinct locations of the text some information on the data is provided, e.g. in the Geostatistical analysis subchapter of the Methods chapter (page 4) says: "COVID-19 cases from March 26th, 2020 to June 25th, 2021 at the FSA (Ontario-wide) and PC-level (for 6 populated areas: Hamilton, Kitchener/Waterloo, London Ottawa, Toronto, and Windsor, including Essex county) were evaluated using ArcGIS Desktop 10.7 [ESRI] with the Geostatistical Analyst extension". It is followed by the first information on the data (page 6, the same subchapter) by indicating that "COVID-19 testing data compiled by ICES contained metadata on tested individuals, such as gender, age, and relevant predicate health conditions", and only later, at the subchapter titled Preparation of Ontario COVID-19 case data from ICES (page 7), we get the introduction of the data source. Somehow the introduction of the data source should be provided more consistently, maybe in a separate chapter before "Methods" titled "Data". The importance of the data for any geostatistical study is essential since the quality and the reliability of the data validates the conclusions.

**Response:**

 We agree that the description of the data source should come before our description of the geostatistical methods used to analyze it. We have reordered the sections “Preparation of Ontario COVID-19 case data from ICES” and “FSA and PC Boundary Files,” which now appear before the “Geostatistical analysis” subsection of the Methods section **.**

**Comment #3:**

 Page 6, Methods: "COVID-19 testing data compiled by ICES contained metadata on tested individuals, such as gender, age, and relevant predicate health conditions." 

 This is the first appearance of the abbreviation ICES, please provide here the full name of the organization!

**Response:**

 ICES was previously an abbreviation for the ‘Institute for Clinical Evaluative Sciences', but is no longer. ICES is now the proper legal name of the organization (see: https://www.ices.on.ca/About-ICES/New-name-and-logo). Our agreement with ICES and representatives from ICES requested that the former abbreviation should not be included in the manuscript.
